# Dynamic Mechanical and Nanofibrous Topological Combinatory Cues Designed for Periodontal Ligament Engineering

**DOI:** 10.1371/journal.pone.0149967

**Published:** 2016-03-18

**Authors:** Joong-Hyun Kim, Min Sil Kang, Mohamed Eltohamy, Tae-Hyun Kim, Hae-Won Kim

**Affiliations:** 1 Institute of Tissue Regeneration Engineering (ITREN), Dankook University, Cheonan, Republic of Korea; 2 Department of Nanobiomedical Science and BK21 PLUS NBM Global Research Center for Regenerative Medicine, Dankook University, Cheonan, Republic of Korea; 3 Department of Biomaterials Science, College of Dentistry, Dankook University, Cheonan, Republic of Korea; Texas A&M University Baylor College of Dentistry, UNITED STATES

## Abstract

Complete reconstruction of damaged periodontal pockets, particularly regeneration of periodontal ligament (PDL) has been a significant challenge in dentistry. Tissue engineering approach utilizing PDL stem cells and scaffolding matrices offers great opportunity to this, and applying physical and mechanical cues mimicking native tissue conditions are of special importance. Here we approach to regenerate periodontal tissues by engineering PDL cells supported on a nanofibrous scaffold under a mechanical-stressed condition. PDL stem cells isolated from rats were seeded on an electrospun polycaprolactone/gelatin directionally-oriented nanofiber membrane and dynamic mechanical stress was applied to the cell/nanofiber construct, providing nanotopological and mechanical combined cues. Cells recognized the nanofiber orientation, aligning in parallel, and the mechanical stress increased the cell alignment. Importantly, the cells cultured on the oriented nanofiber combined with the mechanical stress produced significantly stimulated PDL specific markers, including periostin and tenascin with simultaneous down-regulation of osteogenesis, demonstrating the roles of topological and mechanical cues in altering phenotypic change in PDL cells. Tissue compatibility of the tissue-engineered constructs was confirmed in rat subcutaneous sites. Furthermore, *in vivo* regeneration of PDL and alveolar bone tissues was examined under the rat premaxillary periodontal defect models. The cell/nanofiber constructs engineered under mechanical stress showed sound integration into tissue defects and the regenerated bone volume and area were significantly improved. This study provides an effective tissue engineering approach for periodontal regeneration—culturing PDL stem cells with combinatory cues of oriented nanotopology and dynamic mechanical stretch.

## Introduction

Periodontal disease (periodontitis) and periodontium defects, including irreversible destructions of alveolar bone, periodontal ligament (PDL) and other tissues surrounding and supporting tooth structure, eventually lead to tooth loss [1,2]. Traditional clinical treatments have focused on the removal of contaminants, but the current periodontal therapy has emerged on the regeneration of the damaged tissues in the periodontium. The regeneration process involves a series of interactions between cells comprising the complex periodontium tissue structure, i.e., alveolar bone, root cementum and PDL. Therefore, a complete regeneration of the multiple-domain tissue structure has been difficult to attain, and particularly restoring the native structure of PDL and root cementum has far yet been successful [1,3].

Among the tissues, PDL plays a central role in the regeneration process of periodontal pocket. PDL is a dense connective tissue between the root cementum and the alveolar bone, which is well-organized flexible, fibrous suspension system anchoring the dental root to the surrounding alveolar bone [4,5]. In PDL, an important population of cells has been identified as stem cells which have a multipotency, and the cells are recognized to play essential roles in the regeneration process of the periodontal complex tissues, regulating osseous remodeling and ligament formation by differentiation into either cementoblasts or osteoblasts depending on the needs and conditions [6,7]. Many studies have thus demonstrated the PDL primary cells and the isolated stem cell populations showed osteoblastic differentiation *in vitro* in response to common osteogenic culture conditions [6,8,9].

One important consideration in PDL tissue is that PDL cells are continuously subjected to mechanical stress caused by occlusal forces [2,6], and in response to the mechanical cues the remodeling of the PDL and the neighboring alveolar bone occurs [10–13]. Recent accumulated studies have shown that *in vitro* cultured PDL cells responded sensitively to mechanical stresses in their proliferation and differentiation [14], and the use of mechanical forces has been suggested to stimulate and differentiate PDL cells for tissue engineering purposes [2].

At this point, we aimed to engineer PDL primary cells first by applying mechanical stresses. The mechanical stressed conditions are considered to mimic the native tissue environments, enabling PDL primary cells to recognize the mimicking conditions and to behave properly as if they do within the *in vivo* PDL tissue structure. Together with this mechanical cue, we also provided topological substrate conditions to the PDL cells by culturing the cells upon nanofibrous membranes. Nanofibrous membranes made of poly-caprolactone/gelatin were directionally-oriented to enable PDL primary cells to recognize nanotopological cues underlying and thus to align the cells in one direction, which is also considered to *ex vivo* mimic the arranged ligament tissue structure. The engineered PDL cells under simultaneous topological and mechanical cues were assessed *in vitro* in terms of cell alignment and protein expressions related with PDL extracellular matrix (ECM) components. In fact, some recent studies have examined the effects of mechanical or topological cues on ligament cells including PDL *in vitro* [15–17], however, there is few that addressed the combinatory effects of both cues where biomaterials are also engaged in. Furthermore, we develop the PDL cell-cultured nanofibers into tissue-engineered constructs and address the performance in periodontal defect models *in vivo*, which has not been conducted so far. We consider this work provides an effective and useful PDL tissue engineering methodology by enabling the simultaneously application of physical and mechanical cues that is to mimic the native tissue environments, and hope the method can be applied for engineering other similar tissues.

## Materials and Methods

### Nanofibrous membrane scaffolds

Composite poly-caprolactone (PCL)/gelatin (Gel) nanofiber was prepared using an electrospinning technique. PCL (Mw 80,000, #440744), Gel (type B, bovine skin, #G6650) and tetrafluoroethanol (TFE, #TG3002) were all obtained from Sigma-Aldrich, USA. Both PCL and Gel with a concentration of 15 wt % were prepared by dissolving each in TFE at 50°C for 24 h, and the 1:1 mixture was prepared while vigorously stirring for 2 h. Five milliliter of the solution was electrospun using a 10 ml syringe with a needle diameter of 0.4 mm, at a flow rate 1 ml/h. A high voltage (11.5 kV) was applied to the tip of the needle attached to the syringe when a fluid jet was ejected. To orient the nanofibers in one direction, a rotating cylindrical metal collector was used (15 cm distanced from the needle tip of the needle) which was rotated at a speed of 900 rpm. The electrospun nanofibers were vacuum-dried overnight.

The surface morphology of the electrospun nanofiber was examined by a high resolution scanning electron microscope (SEM; JEOL-JSM 6510). The average nanofiber diameter and nanofiber angle distribution were calculated from the SEM images obtained at random locations. The tensile mechanical properties of the nanofibers were measured by using universal testing machine (Instron 3344) at a cross-head speed of 10 mm/min. Nanofiber membranes were prepared with a dimension of 30 mm x 4 mm x 200 μm (gauge length 10 mm), and a tensile load was applied to obtain a stress-strain curve. Based on this, the elastic modulus, maximum tensile stress, and strain at failure (elongation) were measured. Five specimens were tested for each composition.

### Primary periodontal ligament cell culture from rats

Animal cell primary culture was performed after the approval (No. 13–018) by Dankook University, Institutional Animal Care and Use Committee, Republic of Korea. Rat periodontal ligament (PDL) cells were isolated from 5-week-old male Sprague-Dawley (SD) rat (Daehan Biolink Co., Ltd, Chungbuk, Korea). Briefly, four incisors of SD rat were extracted and PDL tissues were scraped and digested with collagenase type I solution. After culture in normal medium which consisting of α-modified minimal essential medium (α-MEM, SH30265.02, HyClone, USA) supplemented with 10% fetal bovine serum (FBS, SH30919.03, HyClone, USA) and containing 100 U/ml penicillin and 100 mg/ml streptomycin (Pen/Strep, 15140–122, Gibco, USA), cells were gathered by trypsinization (0.05% trypsin-EDTA, 25300–062, Gibco, USA), re-suspended and cultured until they reached near confluence. Cells were cultured with refreshing medium every 2–3 days, and the cells at 3–4 passages were used for further experiments.

### Cell phenotype analysis

Before use, cellular population at 3 passages was analyzed by flow cytometry profile using fluorescence-activated cell sorter (FACSCalibur, BD Biosciences, San Jose, CA). Some characteristic surface markers for multipotent mesenchymal stem cells, including CD44 (sc7946), CD73 (sc14682), CD106 (sc8304), CD146 (ab75769) and TGF-β receptor1 (TGF-βR1) (ab31013) for positive signals and CD31 (sc1506), CD34 (sc7045) for negative signals, were analyzed on the PDL cells. Briefly, single cell suspensions were obtained and fixed. Samples were stained with antibodies of each factor, and FITC-labeled second antibody was also added. The experiment was repeated three times.

### Cell attachment and proliferation

*In vitro* cell attachment behavior and proliferation to the nanofiber membranes was evaluated. Prior to cell tests, samples were prepared and sterilized with ethylene oxide (EO) gas. For the cell attachment and proliferation test, 5 x 10^4^ PDL cells were seeded onto each sample and tested at 1, 3, 7 and 10 days of incubation. Quantification of the cell attachment and proliferation level was assessed by double-stranded DNA (dsDNA) fluorescence kit (PicoGreen quantitation assay kit, Molecular Probe), according to the manufacturer’s instructions. Briefly, the cultured cells were lysated followed by three cycles of freezing/thawing process. Cell lysates were collected by centrifugation at 12,000 rpm at 4°C for 15 min, and the resultant supernatants were used. Fifty microliter of a 1:200 dilution of PicoGreen reagent were added in a 50 μl volume of the cell lysates and incubated for 5 min in the dark. Three samples in each group were used for this assay and read on a multimode microplate plate reader (Spectra Max M2e, Molecular Devices). The data were averaged from duplicate tests. After subtracting the fluorescence value of the reagent blank from that of each of the samples, a standard curve made using a provided dsDNA standard sample, and the intensity of experimental samples was interpolated from this standard curve to determine the cell attachment and proliferation of the sample.

For observation of the cell morphology, the cultured cells were washed and then subsequently fixed. Alexa Fluor 546-conjugated phalloidin (Invitrogen A22283) and ProLong Gold antifade reagent with 4’,6-diamidino-2-phenylindole DAPI (Invitrogen P36935) were used to stain F-actins and nuclei, and the cells were visualized via fluorescence microscope (IX71, Olympus, Tokyo, Japan) equipped with a digital camera.

### Application of mechanical stress to cell/scaffold complex

The application of a mechanical stress was enabled by means of Flexcell system (FX-5000^TM^ TENSION SYSTEM). Flexcell system is commercially available stretching devices, which designed to apply reproducible cyclic tension or compression by changing the magnitude and the frequency for the cells deformation [6,18]. Uniflex culture plate was modified with nanofiber membranes. The nanofiber prepared with a rectangular shape of 32 mm x 15 mm was fixed on the culture plate by using self-leveling silicone glue. Two different nanofiber membranes (either oriented or random) were introduced.

**Fig 1:**
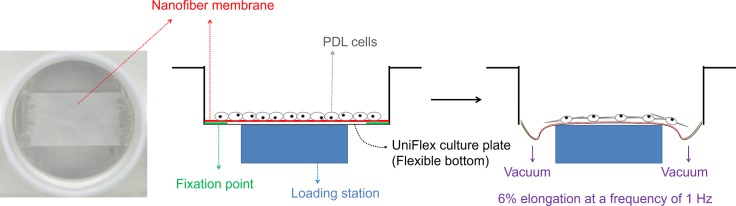
Schematic of experimental design. Mechanical-stressed PDL cells supported on nanotopological-cued nanofiber membrane scaffolds. The Flexcell system equipped with the PDL cells supported on nanofiber matrix, where the dynamic mechanical tensional force was applied to the matrix/cell through equipment vacuum.

The well plates were sterilized with EO gas, and the PDL cells were plated onto the nanofiber-equipped culture plates at a density of 1 x 10^5^ cells per membranes for morphological analyses and 5 x 10^5^ cells per membranes for other *in vitro* and *in vivo* tests. After culturing of the cells for 2 days, the experimental groups were divided into four; Group ‘DA’: dynamic cell culture on aligned nanofiber, Group ‘SA’: static cell culture on aligned nanofiber, Group ‘DR’: dynamic cell culture on random nanofiber, and Group ‘SR’: static cell culture on random nanofiber. As illustrated in Fig 1, dynamic culture involved applying dynamic strain change of 6% elongation at a frequency of 1 Hz to the cell/scaffold constructs using Flexcell system to provide bilateral mechanical tensile stress dynamically.

For the aligned nanofiber, the nanofiber orientation was set in parallel to the strain direction. The nanofiber orientation with respect to the direction of the mechanical stress was recorded; 0° angle is parallel to the strain and 90° angle is perpendicular to the strain. After the nanofiber orientation angles were determined, the angles were binned into 9 groups for every 10°: 0–10°, 11–20°, …, 71–80°, and 81–90°. Total nanofiber number counted (frequency) was set as 100% and the frequency in each group was expressed relative to the total nanofiber number.

### Cell morphological analyses

For the observation of cell distribution and orientation on the samples, confocal laser scanning microscopy (CLSM; LSM 510, Carl Zeiss) was used. Each test was performed on three replicate samples (n = 3). The cells grown on each sample were fixed, and Alexa Fluor 546 phalloidin and DAPI were treated to stain F-actins and nuclei, respectively. Based on the stains, cytoplasmic protrusion and orientation of the cell were analyzed. The number of cytoplasmic protrusions of each cell was quantified and average length of each cytoplasmic protrusion was also determined. The type of cytoplasmic protrusions was also determined unipolar, bipolar, or multipolar, and particularly bipolar cells were considered of importance as they recognize the bilateral stress and alignment of the underlying nanofiber substrate. Furthermore, the orientation angle of a cell was measured as the absolute value (in degree) of the smallest angle between the major axis of the cell and the direction of the nanofiber using image analysis software (Scion image, National Institutes of Health, USA), and results give values which vary between 0° (parallel to strain) and 90° (perpendicular to strain).

The cell morphology on the nanofiber membranes was also observed by SEM. For this, the cells cultured on the nanofibers were fixed with 4% paraformaldehyde and dehydrated with a graded series of ethanol (75, 90 and 100%) each for 10 min. After treatment with hexamethyldisilazane, the samples were sputter-coated with carbon, and then visualized using a JEOL-JSM 6510 at an accelerating voltage of 15 kV.

### Alkaline phosphatase determination

The enzymatic activity of alkaline phosphatase (ALP) was determined to check osteogenic differentiation of PDL cells during the different culture conditions (DA, SA, DR, and SR). After culture, cell lysates from each sample were achieved by freeze-thaw cycles three times and followed by addition of cell lysis buffer. The samples lysates were moved to the tubes and then centrifuged, and the collected cell lysates were used for measurements. The samples (50 μl) were added to 50 μl of working reagent containing equal parts (1:1:1) of 1.5 M 2-amino-2 methyl-1-propanol (Sigma, A5888), 20 mM p-nitrophenol phosphate (Sigma, P4744), and 1 mM magnesium chloride (Sigma, 208337). The samples were then incubated, and the reaction was stopped with the addition of 100 μl of 1 N NaOH on ice. The *p*-nitrophenol produced by the reaction was then determined based on the absorbance at 405 nm using micoplate-reader (iMark, BioRad, CA, US). The reading for the sample was calculated from a standard ALP activity curve prepared using *p*-nitrophenol stock standard (Sigma, N7660). Cell proliferation was measured by qualify dsDNA via PicoGreen fluorescence assay to standardize ALP production measurements. The ALP specific activity was calculated by normalizing the ALP activity to dsDNA produced from each sample. Each test was performed on three replicate samples (n = 3).

### Expression of proteins related with PDL

The production of periostin, tenascin and TGF-β by the PDL cells, when affected by the different culture conditions (DA, SA, DR, and SR), were measured by an ELISA. A 100 μl cell lysate was placed into a 96-well plate, and incubated at 4°C overnight. After washing the sample twice, 200 μl blocking buffer (5% nonfat dry milk in PBS) was added and the plate was incubated for 2 h at room temperature. After washing twice with PBS, periostin (sc67233), tenascin (sc9871) or TGF-β (ab66043) primary antibody was added to each well and incubated at 4°C overnight. After washing 4 times with PBS, conjugated secondary antibody was added and incubated for 1–2 h at room temperature. After washing, TMB solution was added to the wells and incubated for 30 min at room temperature; this step was followed by the addition of a stop solution (2 M H_2_SO_4_). The absorbance was measured at 450 nm.

### Animals and *in vivo* design

The protocol of the housing, care and experimental protocol (No. 12–027) were approved by Dankook University, Institutional Animal Care and Use Committee, Republic of Korea. A total of eighteen, 11 weeks old, 350–400 g healthy male SD rats were included in this study.

As the animal models, first subcutaneous model was used to determine tissue reactions of the cell/scaffold tissue-engineered samples (DA, SA, DR, and SR groups). Next, bilateral premaxillary periodontal defect model (4 mm in diameter) was designed to investigate the regeneration of PDL and surrounding tissues by the implanted tissue-engineered constructs. In particular, two differently-conditioned PDL tissues were introduced; either sound or removed. Thus, the experimental groups for these PDL *in vivo* models include DA implanted in sound PDL tissue ‘DA (sound)’, SA implanted in sound PDL tissue ‘SA (sound), DA implanted in removed PDL tissue ‘DA (remov), and SA implanted in removed PDL tissue ‘SA (remov)’, and two control groups (only aligned nanofiber in removed PDL and empty removed control).

### *In vivo* study in subcutaneous tissue

The tissue responses of the experimental samples were investigated in rat subcutaneous tissue. Four SD rats were anesthetized by injection of 80 mg/kg Zoletil^®^ and 10 mg/kg Rompun^®^. The skin on the dorsal region of the rat was shaved, and disinfected. A skin incision was made, and four small subcutaneous pockets were formed by blunt dissection on the backside laterally from the spine of each rat. Samples were then placed into the prepared area away from the incision site and the incision was subsequently sutured with 4–0 non-absorbable monofilament suture material (Prolene^®^, Ethicon). Four samples were implanted for each membranes group. Following recovery from anesthesia, the rats were housed individually and standard pellet food and water were provided *ad libitum*. At 4 weeks after operation, the animals were sacrificed.

### *In vivo* study in premaxillary periodontal defects

During the surgical operation, the animals were anesthetized with injections of 80 mg/kg Zoletil^®^ and 10 mg/kg Rompun^®^. 0.5% lidocaine with epinephrine was locally injected into gingival tissues of premaxilla to decrease pain and bleeding during operation. Animals were placed in dorsal recumbency and operation field was prepared in the standard manner for aseptic surgery serial using 10% povidone iodine and 70% ethyl-alcohol. All instruments were sterilized prior to surgical procedures and all the procedures on animals were carried out using sterile techniques. Following an incision through the palatal gingival epithelium, the underlying maxillary alveolar bone of premaxilla was exposed. Two defects were created on 14 animals, and total 28 defects were randomized to 6 groups. Defect model creation initially involves the extraction of tooth, after which the PDL and germ tissues were either removed completely (‘removed’ model) or remained intact (‘sound’ model). 4 mm diameter trephine bur was used to create a standard full-thickness round defect on the lateral surface of each premaxilla bone. The thickness of the defect was approximately 200 μm at the center. During creation of the defect, the surgical field was continuously irrigated with cooled sterile isotonic saline. After this treatment, the extracted incisors were replaced into the extraction socket. Subsequently, membranes were trimmed to extend about 2 mm outside the margins of the defect and the defect was covered. The defect was either left empty as a control. After the delivery of samples, the mucosal flaps were carefully repositioned on the outer side of the maxilla and sutured with simple interrupt suture pattern using 4–0 absorbable suture material (Vicryl^®^, Ethicon).

After surgery, the rats were housed individually in cage under conditions at 12-h light/dark cycle, with relative humidity and temperature controlled environment. Animals were maintained on a grinded pellet food with various sizes of diet and water *ad libitum*. Animals were monitored for signs of infection, inflammation and adverse effects by visual observation during study periods. The animals were sacrificed for harvesting of the samples and surrounding tissues at 4 weeks after surgery.

### Micro-computed tomographic analysis

At 4 weeks after surgery, animals were sacrificed by CO_2_ inhalation and cervical dislocation, and the premaxillary defects area was explanted and the implants with surrounding tissue were retrieved for micro computed tomography (μCT) imaging and histological analysis. The specimens were fixed in 10% neutral buffered formaldehyde solution, and imaged using an *in-vivo* high resolution μCT system (Skyscan 1176, Skyscan, Aartselaar, Belgium) to evaluate tissue recovery and bone regeneration. The harvested samples were scanned with a camera pixel size of 12.56 μm, and a frame averaging of three was employed together with a filter of 1 mm aluminum, a rotation step of 0.5°, the rotation angle was 180°. The X-ray tube voltage was 65 kV and the current was 385 μA, with an exposure time of 279 ms. Serial coronally oriented tomograms were reconstructed from the raw images in the NRecon Skyscan reconstruction software. Region of interest (ROI) was precisely positioned over the each defect for quantitative analyses, encompassing all new bone within the defect site. Reconstructed images over the ROIs using CTAn Skyscan software were used to analyze bone formation and three-dimensional (3D) images were created. Three samples from each group were measured for μCT imaging to qualitatively visualize implant location and bone formation, and the amount of defect healing was expressed as the percentage of new bone volume (%), bone surface (mm^2^), and bone surface density (1/mm).

### Histological observation

After μCT imaging, harvested samples were prepared for histological analysis. For this, fixed specimens were decalcified with RapidCal^TM^ solution (BBC Chemical Co., Stanwood, WA, USA), and dehydrated in a graded series of increasing ethanol dilutions (from 70% to 100%). To obtain thin sections, samples were bisected in the middle of the defect and embedded in paraffin. The blocks were serially cut into 5 μm thick sections using a microtome (RM2245, Leica, Germany), and histological preparations were prepared. The sections were placed on slide glass and stained with hematoxylin-eosin (HE) and Masson’s trichrome (MT) stain using standard techniques to check biocompatibility and periodontal tissue regeneration. Under a light microscopy (IX71, Olympus, Tokyo, Japan), digital images were photographed using Meta-Morph, and the results of periodontal tissue regeneration within the defect were presented.

### Statistical analysis

All data were presented as mean ± standard deviation. Differences between groups were assessed by one-way analysis of variance (ANOVA) or student t-test. For ANOVA, Bonferroni correction was used as a post-hoc analysis. *P* value < 0.05 was considered significant.

## Results

### Nanofibrous membrane scaffolds

**Fig 2:**
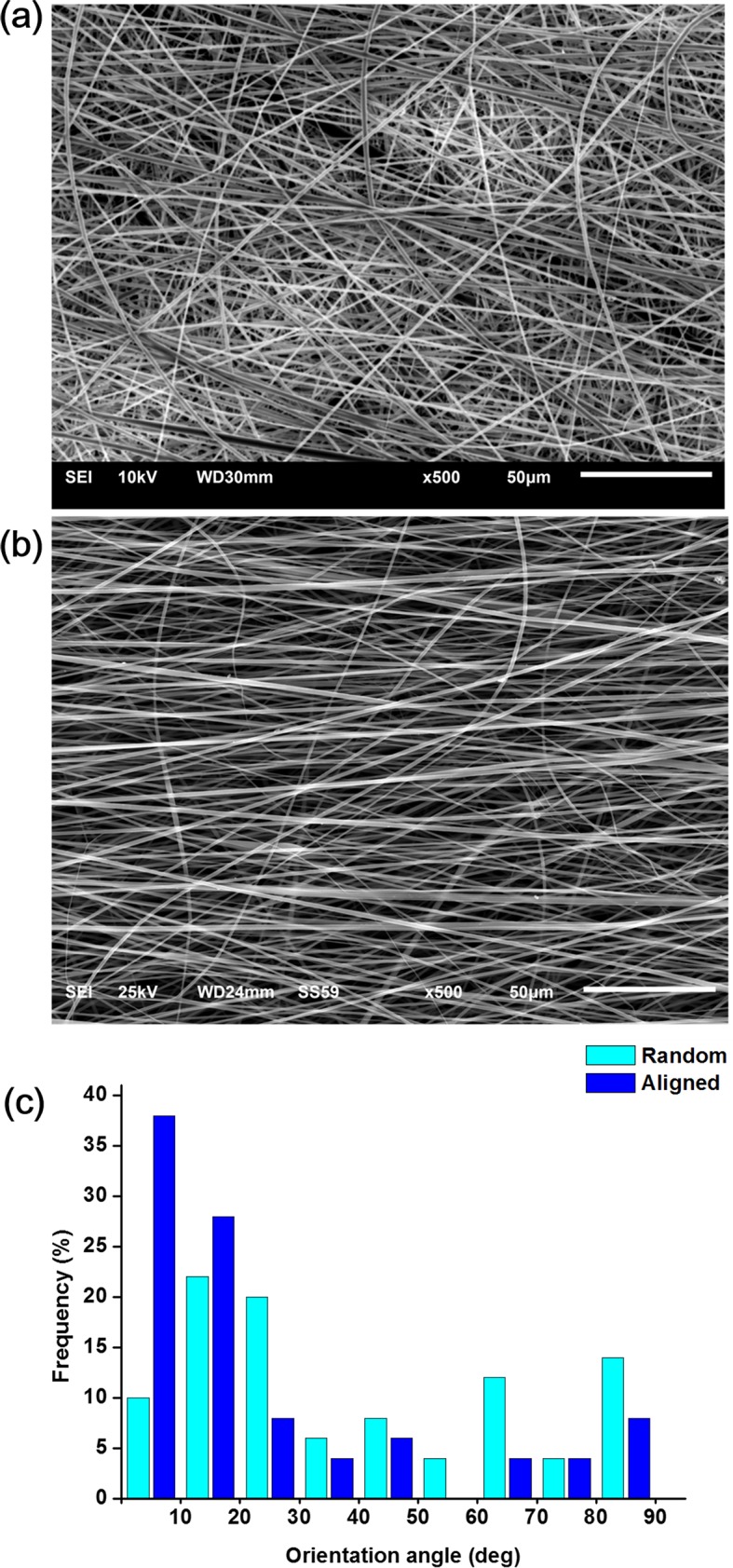
Surface morphology of the membranes. Electrospun membranes made either random or oriented to provide different nanotopological alignment cues to PDL cells. SEM images of the (a) random and (b) aligned nanofibers. (c) Histograms of the orientation of the nanofibers.

Surface morphology of the membranes with two different alignment conditions was examined by SEM. Membranes with the randomly distributed fibers were shown (Fig 2A), which in contrast to well aligned membrane (Fig 2B). Histograms of the orientation of the aligned and random fibers are also presented (Fig 2C). Although the aligned nanofiber was contrasted to the random nanofiber, a fraction of the nanofibers was also randomly oriented due to the limitation in nanofiber processing. The mechanical properties of the nanofibers including elastic modulus, maximum stress recorded, and strain at failure (elongation) are summarized in S1 Table. While the elastic modulus was higher in the aligned nanofiber, the maximum stress and elongation were higher in the random nanofiber.

### Stem cell adhesion and growth on nanofibers

Prior to the cell study on the nanofibers, some representative surface markers of multipotent MSCs were examined for the of PDL cells using FACS analysis. Results showed the PDL cells expressed high levels of positive signals, including CD44 (74.71%), CD73 (66.68%), CD106 (92.12%), CD146 (91.76%) and TGF-βR1 (90.23%), whereas expressed low levels of negative signals, including CD31 (0.63%) and CD34 (0.75%), which indicates the PDL cells have characteristics of multipotent MSCs.

**Fig 3:**
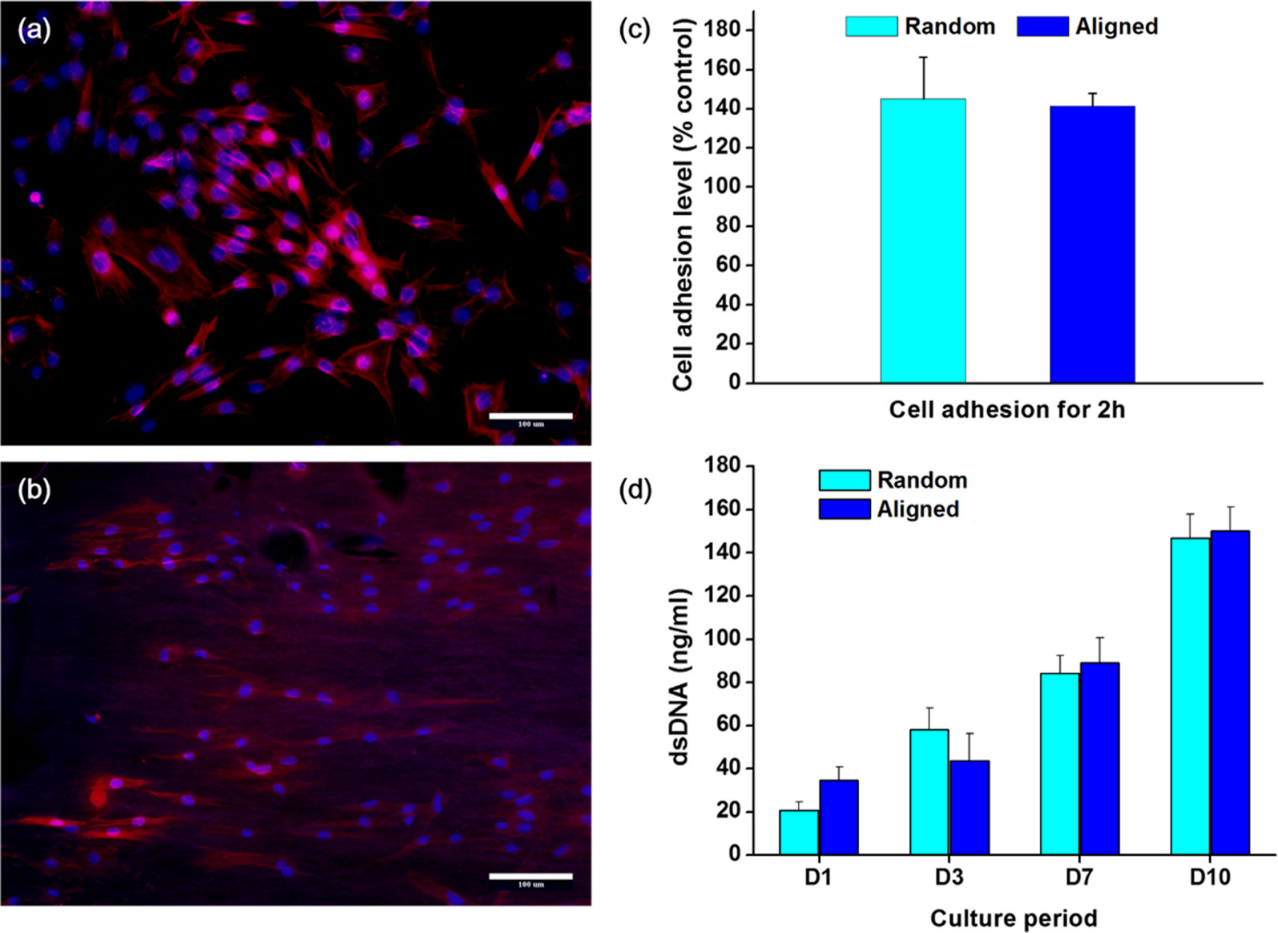
Effects of the nanofiber membrane on the initial cellular adhesion behavior. Fluorescent images of cell attachment on (a) random and (b) aligned nanofiber. PDL cells recognize the underlying nanofiber alignment, conforming the shape of cell spreading to the nanofiber orientation (Magnification x200, scale bar 100 μm). (c) Cell attachment level, and (d) subsequent proliferation (*p < 0.05, by student *t*-test).

Next, the effects of the nanofiber membrane on the initial cellular adhesion behavior were examined. The PDL cells, which normally have typical fibroblast-like appearance, extended and aligned to the direction of nanofiber orientation with a spindle-like shape after 12 h of culture. On the randomly distributed nanofiber membrane PDL cells also attached randomly with a polygonal shape and showed cytoskeleton extensions to multiple directions (Fig 3A). On the other hand, on the aligned nanofiber, the PDL cells aligned almost parallel to the direction of fibers (Fig 3B). The initial cell adhesion level was quantified (Fig 3C). The cell adhesion level, normalized to that of control culture dish, was higher on both nanofiber types, and between the two groups there was no significant difference. The cell proliferation level was then recorded for up to 10 days by means of dsDNA quantification (Fig 3D). There was no significant difference between the groups.

### Effects of cyclic loading and nanofiber alignment on cell orientation and protrusion

**Fig 4:**
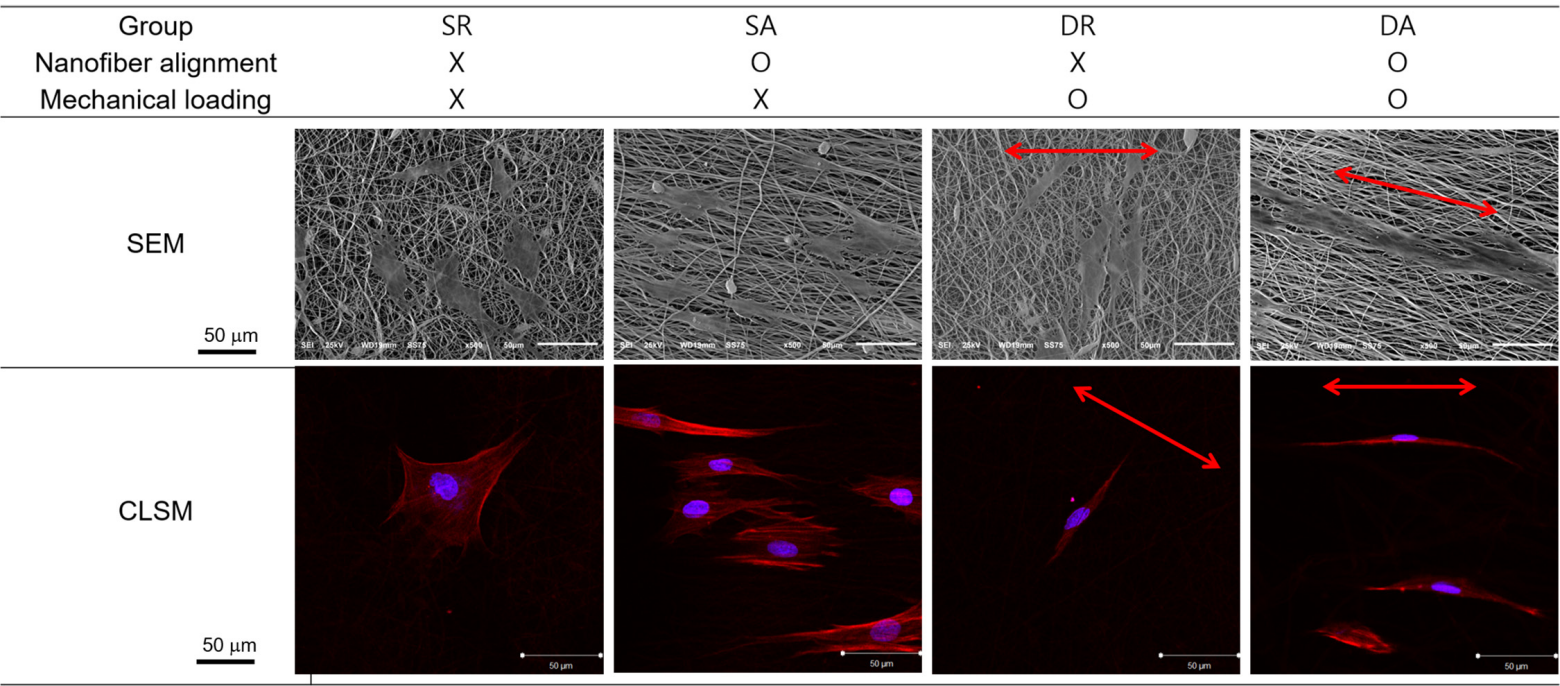
Effect of the cyclic uniaxial stretch on the orientation of PDL cells. Examination of cell shaping on the differently-aligned nanofibers with or without applying dynamic mechanical load. F-actins visualized with rhodamine-phalloidin (red) and nuclei stained with DAPI (cyan) for fluorescent images. Red arrows indicate stretch direction.

Based on attachment and proliferation results, we examined the cell behaviors on the nanofibers under the cyclic loading. The effect of the cyclic uniaxial stretch on the orientation of PDL cells was visualized by SEM and CLSM (Fig 4). Under static condition (no mechanical loading), the cells were randomly spreading on the random nanofibers (SR) whilst the cells were aligned spreading on the aligned nanofibers (SA). Under the cyclic loading condition, cells on the random nanofiber aligned perpendicular to the loading axis (DR, note arrow indicating loading direction). Interestingly, however, the cells on the aligned nanofiber aligned along the loading axis, which was also parallel to the orientation of the nanofibers (DA). The cell shape became quite bi-polar and the elongation extend appeared to be more substantial than the other conditions.

**Fig 5:**
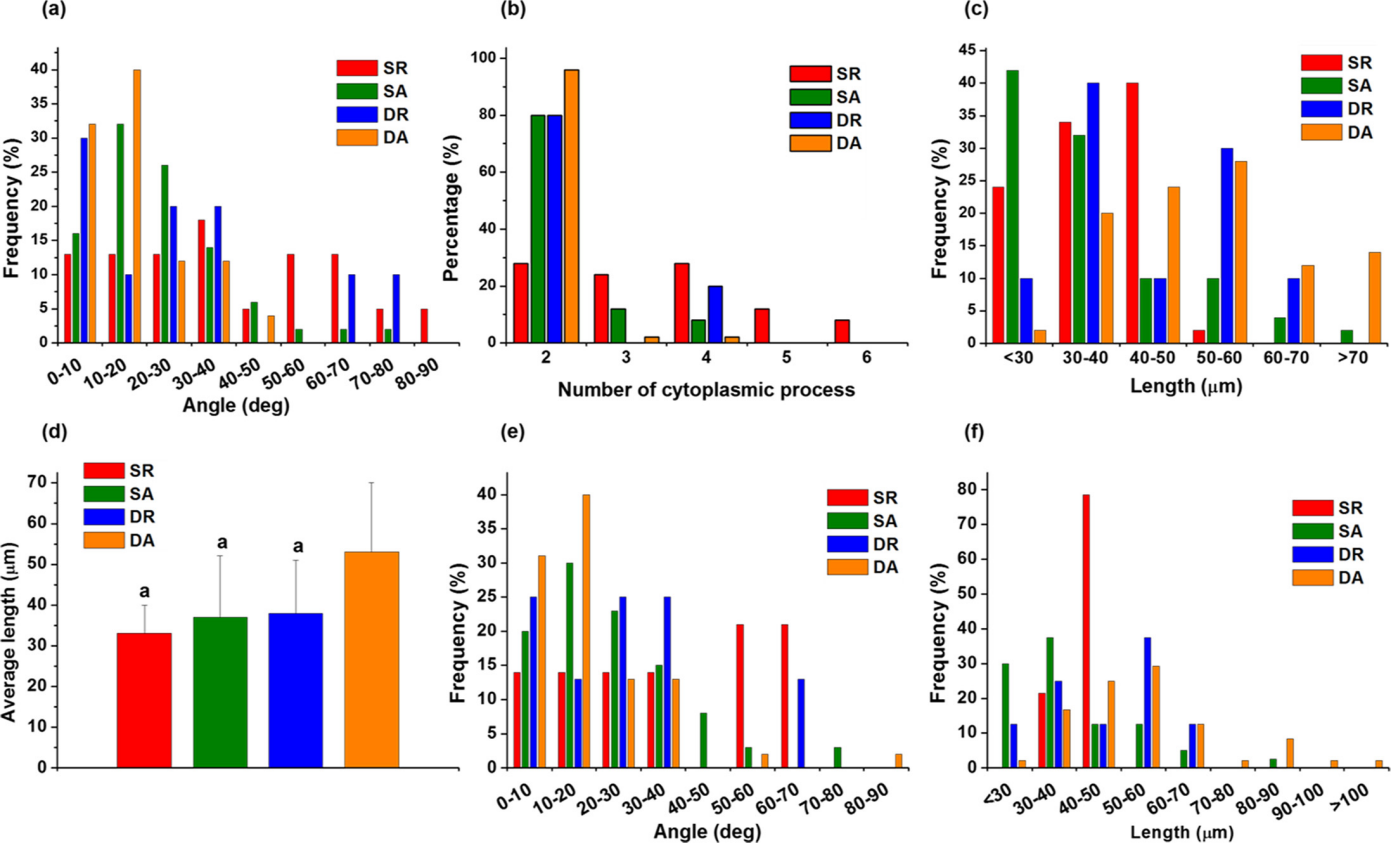
The orientation of and protrusion behaviors of the cells. Analyses of the PDL cell orientation and cytoskeletal protrusion, after cell culture under the influence of nanofiber alignment and/or cyclic mechanical load. (a) Distribution of cell orientation angles. (b) Number of cytoskeletal protrusions, and the (c) distribution of protrusion length and (d) average protrusion length. Only bipolar cells were also analyzed in terms of distribution of (e) orientation angle and (f) protrusion length. (^a^*p* < 0.05 compared to DA, by ANOVA).

The orientation of and protrusion behaviors of the cells were subsequently analyzed. First, the cell orientation angle distribution showed while most cells in ‘DA’ group oriented in single direction those in ‘SR’ group randomly oriented, and the degree of orientation was in the order; DA > SA ~ DR > SR (Fig 5A). The cell protrusion behaviors were also examined. The protrusion analysis showed most cells in ‘DA’ were bipolar (~96%), and also for ‘SA’ and ‘DR’ groups also showed higher level of bipolar cells (~80%), on the other hand, the cells in ‘SR’ group were diverse in cytoplasmic extensions (Fig 5B). Along with the cell protrusion number, the cell protrusion length was also compared. The cells in ‘DA”, ‘SA’ and ‘DR’ groups were shown to have longer protrusion length than ‘SR’ group (Fig 5C). The cells in ‘DA’ group were distributed to have significantly longer protrusion length than the others (Fig 5D). Apart from the total cell analysis, the bipolar cells were also specifically analyzed, first with bipolar cell orientation. The result showed to be similar to that observed in the total cell population; in the order of orientation DA > SA ~ DR > SR (Fig 5E). Furthermore, the bipolar cell protrusion length analysis showed the cells were distributed in the highest length region for ‘DA’, followed by medium length for ‘SA’ and ‘DR’, and the lowest in ‘SR’ (Fig 5F).

### Observation of cell differentiation

**Fig 6:**
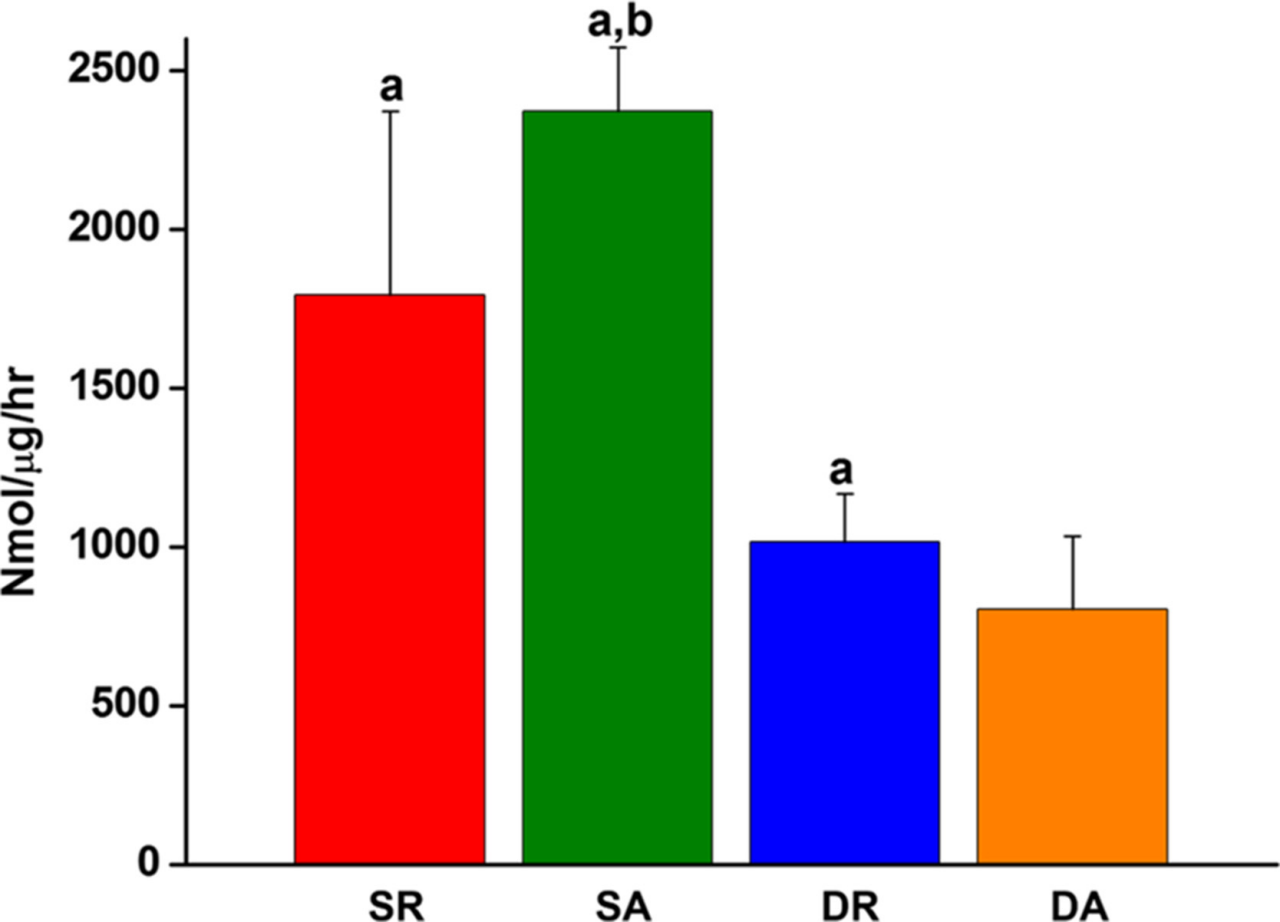
Effects of the nanofiber alignment and cyclic loading on the osteogenic differentiation of PDL cells. The effects were assessed by the ALP activity, an early osteogenic differentiation marker (^a^*p* < 0.05 compared to DA, ^b^*p* < 0.05 compared to DR, by ANOVA).

The effects of the nanofiber alignment and cyclic loading on the differentiation of cells were examined. First, the expression of representative osteogenic factor, ALP, was observed. When the enzymatic ALP activity of the PDL cells was normalized to the dsDNA, the cyclic loaded cells cultured both on the aligned and random nanofibers showed significantly lower ALP than those cultured under static conditions (DR ~ SR > DA ~ SA, Fig 6).

**Fig 7:**
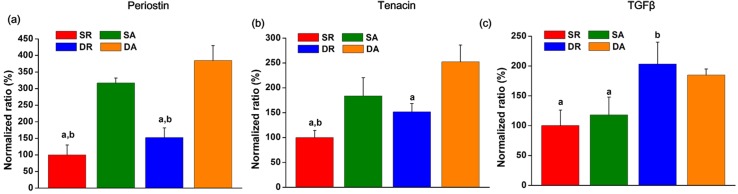
Expressions of proteins related with ligamentogenesis of the PDL cells, as analyzed by an ELISA. (a) Periostin, (b) tenascin, and (c) TGF-β. Results presented when normalized to the static condition with random nanofiber. (^a^*p* < 0.05 compared to DA, ^b^*p* < 0.05 compared to SA, by ANOVA).

Next, the protein expression levels of PDL markers, including periostin, tenascin and TGF-β, were examined by ELISA. The protein expression of each study group was normalized to the SR group. The expression of periostin and tenascin showed a marked difference in the order; DA > SA > DR > SR (Fig 7A & 7B). On the other hand, TGF-β1 expression was significantly improved only by the mechanical stimulus without being affected by the nanofiber alignment; DA ~ DR > SA ~ SR (Fig 7C).

### *In vivo* tissue compatibility

**Fig 8:**
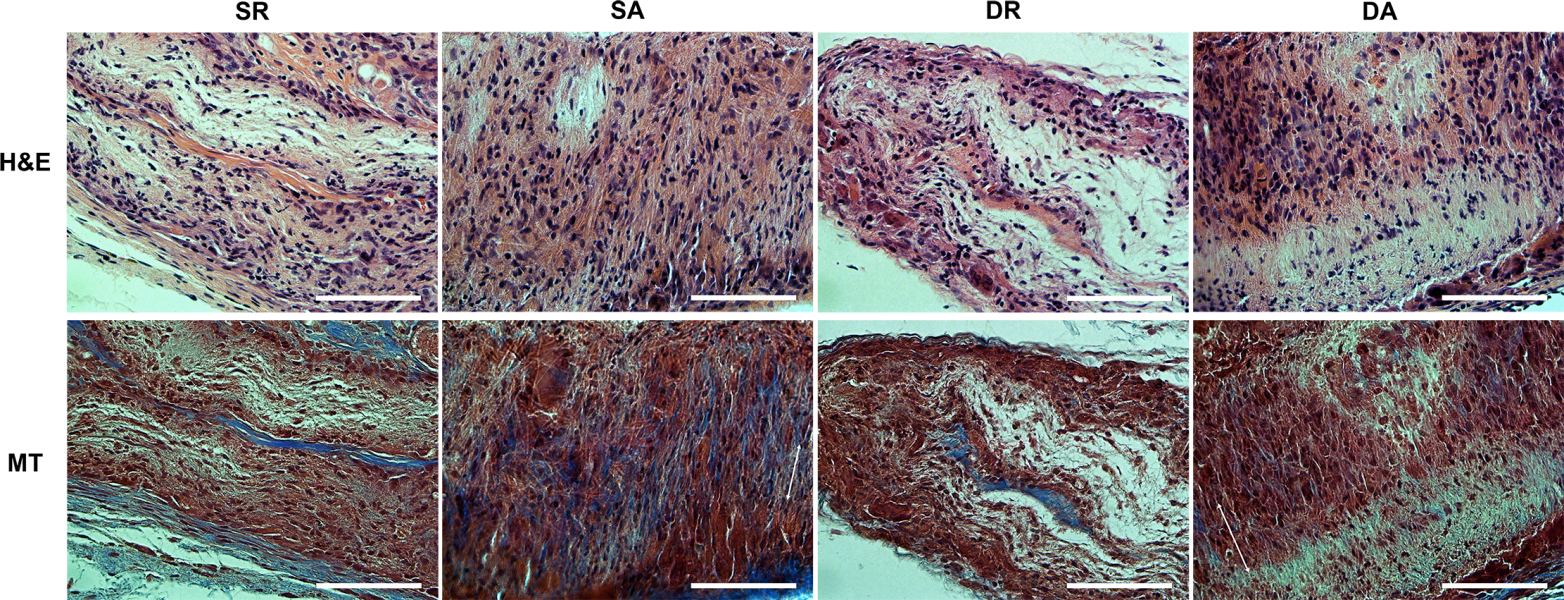
Tissue compatibility of the PDL cell/nanofiber constructs implanted in rat subcutaneous model for 4 weeks. Histological images of HE and MT stains. Notable observation of PDL-like tissues with spindle-shaped oriented cells in the SA and DA groups, as revealed by MT stain. Direction of the PDL cells (two headed arrow) with randomly distributed collagen fibers were marked (Magnification x400, scale bar 100 μm).

The in vivo tissue responses of the PDL cell engineered nanofiber were first assessed in rat subcutaneously. The histological examination showed that all implanted groups were biocompatible (Fig 8). Immune reactions or tissue rejections were not detected in all samples. Many fibroblastic cells were found within the collagenous tissue networks which replaced the original nanofiber-cell constructs. Neovascularization was also shown within the implanted area for all groups. While the collagen fibers were not oriented in the groups engineered on the aligned nanofibers, the PDL cells were well aligned with the implanted membrane matrix (DA and SA), those engineering on the random nanofibers (DR and SR) appeared to be not oriented. The DR and SR groups showed higher amount of PDL-like collagen fibers.

### Tissue regeneration in periodontal defect models

**Fig 9:**
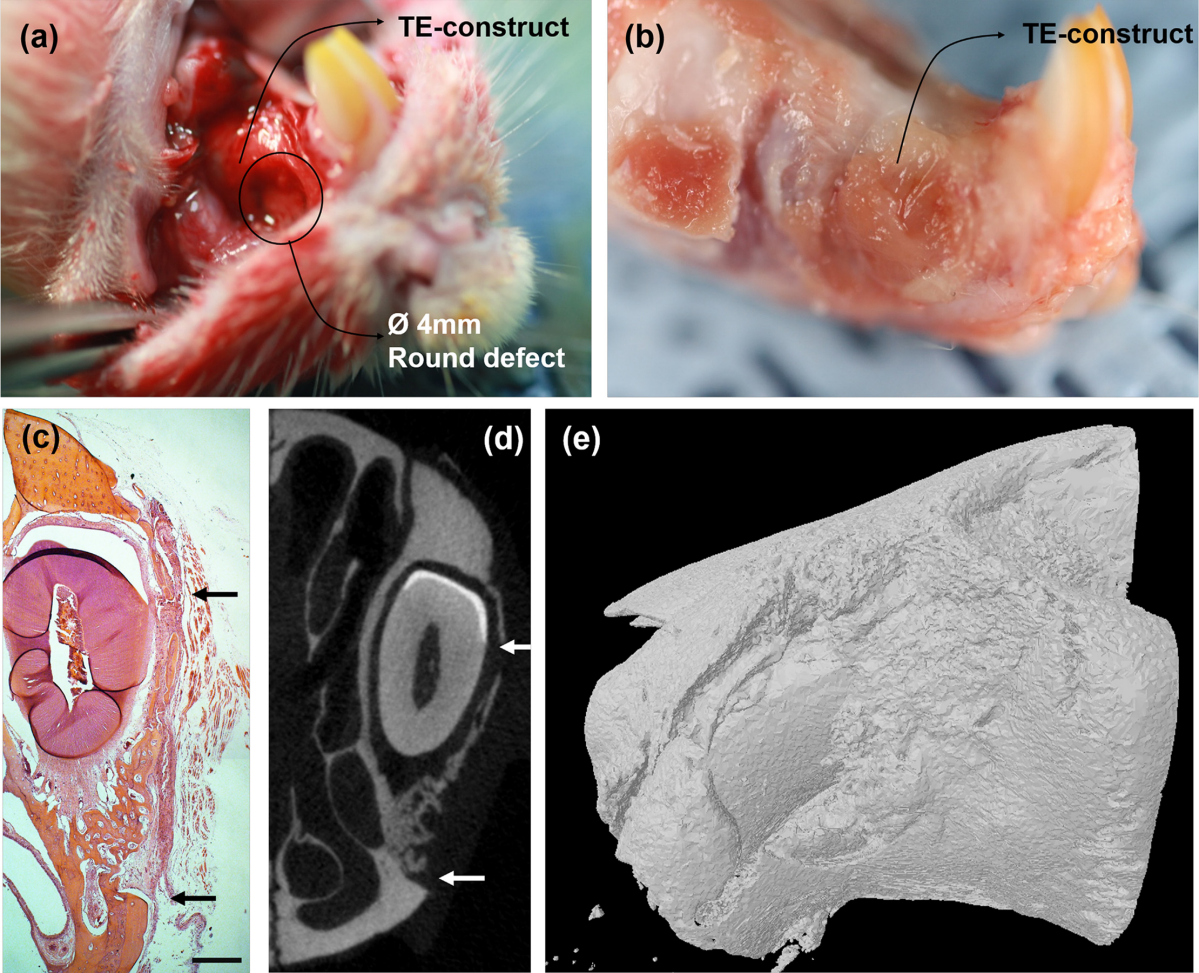
Illustrative images showing the PDL defect models used in this study. (a) Photograph of rat premaxillary operation field. Note the dimensions of the defect used to produce standardized 4 mm diameters round full-thickness defects on the lateral surface of premaxilla bone. Two defects were created on one animal and were covered with tissue-engineered construct. (b) Harvested specimens of rat premaxillary operation field after sacrifice. (c) Representative histology image of HE staining of new bone tissue formed within the defect at 4 weeks (black arrow: defect margins) (Magnification x40, scale bar 500 μm). (d) 2D and (e) 3D μCT images. The original outline of the 4 mm defect is clear (white arrow).

Based on the tissue compatibility study in subcutaneous tissues, we next examined the in vivo efficacy of the PDL cell/nanofiber tissue-engineered constructs for periodontal defect repair and regeneration. Only cells cultured on aligned nanofibers (DA and SA groups) were considered for this in vivo study. A model is explained in Fig 8. Tissue-engineered construct was covered on the 4 mm diameter defect on the maxillary PDL (Fig 9A), and after 4 weeks of tissue healing (Fig 9B), the samples were harvested for histological findings (Fig 9C), μCT imaging and analyses (Fig 9D & 9E). The growth of the maxillary incisors was not interrupted by procedures throughout the study period, except some animals with pulp tissues removed. All animals showed normal activity within 1 day after operation, a good healing response without adverse tissue reactions, and gained weight. The implanted areas remained stable and no visible fibrous invasion and signs of internal inflammation were detected on harvested tissues.

**Fig 10:**
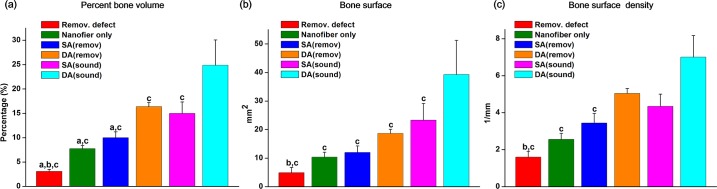
Micro-CT image analyses results of bone regeneration. (a) % bone volume (b) bone surface, and (c) bone surface density. The graph represented statistically significant differences among the study groups on the quantification of new bone formation in premaxillary defects after 4 weeks of healing. (^a^*p* < 0.05 compared to DA (remov); ^b^*p* < 0.05 compared to SA (remov); ^c^*p* < 0.05 compared to DA (sound), by ANOVA).

Morphometric analyses of the μCT images gave quantification of % bone volume, bone surface are, and bone surface density (Fig 10). Compared to the low level of those indices of bone quantity and quality in the PDL-removed defect group used as the negative control, the aligned nanofiber only group was effective in increasing all the values. When DA and SA groups were implanted in the PDL-removed defect, the values further significantly increased. Comparing DA and SA groups, DA group also showed significant difference. When the tissue-engineered groups were implanted in sound PDL model, the values of those bone repair indices were much higher, and the more increase was observed in DA implantation, being in the order; DA (sound) > SA (sound) ~ DA (removed) > SA (removed) > nanofiber only > removal defect.

**Fig 11:**
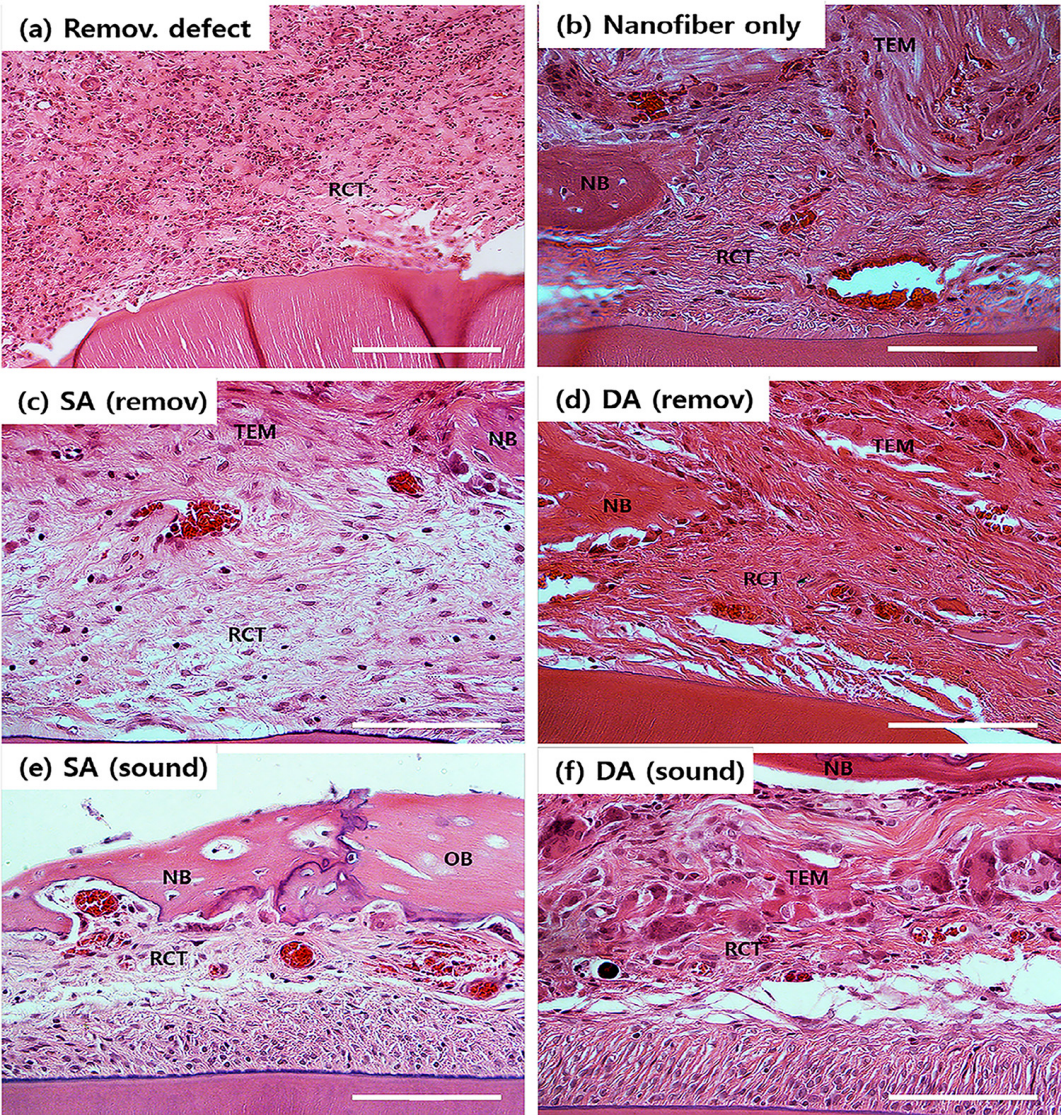
HE stained histological images, showing the tissue regeneration in the periodontal defect model. (Magnification x400, scale bar 100 μm, NB: new bone, OB: old bone, TEM: tissue-e engineered matrix, RCT: reconstructed connective tissue).

Histopathological microscopes of the HE stained samples were examined around the tissue-engineered construct and defect areas (Fig 11). Empty defect group showed the formation of very thin and loose connective tissues covering defect area with very limited new bone formation which was only at the edge of the defect (Fig 11A). Compared to the control group, all other implantation groups showed enhanced bone regeneration with varying degrees. For nanofiber only group, with better bone formation was evidenced than control, but the loose connective tissues were still found profoundly (Fig 11B). For the tissue-engineered groups implanted in PDL-removed, SA (removed) and DA (removed), there was considerably improved new bone formation, which appeared to begin at the caudal edge of the defect area covered by the PDL cell/nanofiber construct (Fig 11C & 11D). New bone tissues were evident in the caudal defect margin, indicating effective roles in guided bone regeneration and osteoconduction. When implanted in sound-PDL defect, the SA (sound) and DA (sound) groups showed intact PDL remained on the surface of rat incisors (Fig 11E & 11F). The newly formed bone was mature and compact with osteoblasts lining. The implanted area was in direct contact with the original PDL tissue without the interposition of connective tissues.

## Discussion

Age-related damages and diseases in periodontal pockets, including alveolar bone, cementum and PDL tissues, require substantial clinical interventions and therapeutic approaches to completely repair. Apart from the hard tissues (bone and cementum), the major soft tissue, PDL, is significantly challenging to recover the structure and biological functions. In fact, in order to enable complete PDL attachment to tooth structure, it is necessary to regenerate root cementum, alveolar bone and intervening PDL in a coordinated fashion [1]. However, current therapeutic technique cannot achieve complete regeneration of periodontal tissues, and new approaches and materials have thus been significantly searched [19].

Stem cell-based tissue engineering that utilizes proper scaffolding conditions is considered promising approach to the regeneration of periodontal tissues. In order to realize this, it is important to recognize the ECM conditions that the stem cells reside and the mechanical and physical environments that provide appropriate cues for the maintenance and differentiation of stem cells to target lineage tissues.

Here we used nanofibrous matrix for the culture of PDL cells and applied cyclic mechanical load to the matrix-cell construct, aiming to provide nanotopological and mechanical cues to the cells that drive their subsequent roles in the PDL regenerative processes. As the topological cues, alignment of nanofibers was also considered. Not only for the mechanical merits, but have the aligned nanofibers shown to improve the cellular orientation [20,21]. The ECM of PDL is also highly organized aligned matrix of collagenous fibers, thus orienting cellular alignment on nanofibrous matrix can be a powerful approach for regenerating PDL [22].

Along with the topological cue, mechanical stimulus was dynamically applied to the PDL cell-nanofiber matrix by means of Flexcell design. Flexcell has been powerfully used to investigate cellular responses *in vitro* in culture dish under cyclic mechanical tensile forces [23–25]. Here we, for the first time, strategically modified the design by coherently adhering nanofiber mesh on the Flexcell dish surface, informing a novel concept of using artificial matrices with Flexcell mechanical culture system. It is known that occlusal and orthodontic mechanical forces modulate the biological response of PDL, such as cell proliferation, differentiation, ECM synthesis and degradation, and the type, frequency, strength and duration of this stress affect these cellular responses [14,26]. 50-100N occlusal forces were generally measured during chewing movements in several *in vivo* studies, and a vertical load of 100 N of a moderate orthodontic force would correspond to approximately 40 kPa [27]. Here we applied 6% strain, which corresponding to ~36 kPa, to our PDL cell/nanofiber matrix with the Flexcell system, and this applied force is considered to be within the previously reported physiological range of mechanical force.

The nanofiber alignment was first shown to guide PDL cell spreading and alignment. When cells are sensing the surrounding area, they expand and retract cytoplasmic protrusions as if they were probing [11]. The PDL cells as well were shown to exert their alignment along the nanofiber directions, thus conforming to bipolar oriented cells on aligned nanofibers whilst multiple-cytoskeletal extensions were widespread on the random nanofibers.

Interesting finding was the cellular alignment behaviors under the cyclic loading. The PDL cells were perpendicularly aligned on the random nanofiber under the loading, conforming their shape from non-directional to directional (bipolar), and this behavior has also been noticed elsewhere [18]. The cells seemed to sense the iso-stress lines generated by the mechanical field, and then extend their probes along the lines, shaping their cytoskeletal processes accordingly. Previously, several researchers have also observed the phenomenon of re-alignment of PDL cells under stretch conditions *in vitro* [6,18,28]. In this study of using aligned nanofiber, PDL cells became parallel to the nanofiber orientation and the cellular elongation was more conspicuous than that noticed in the random nanofiber. In this study, the cellular probing may prefer the continuum mass, i.e., single nanofiber line—which is considered the shortest probing distance—rather than crossing the barrier of nanofiber interspacing. Therefore, the cellular orientation differed under the cyclic loading depending on the nanofiber alignment is considered to result from a combined cellular sensing/probing of mechanical stress distribution and external topological cues. In terms of the degree of cellular orientation or bipolar protrusion, DA was the highest, DR and SA were in the middle, and SR was the lowest, demonstrating dynamic mechanical loading and nanofiber alignment played a synergistic role in this behavior. Such significantly altered cellular alignment and shape can significantly influence the subsequent matrix synthesis and differentiation [29,30].

Another important in vitro outcome influenced by the nanotopological and mechanical cues was demonstrated on the PDL cell differentiation behaviors [31]. PDL stem cells are known to play a decisive role in maintaining the balanced metabolism between hard and soft tissue turnover by stimuli in both osteo- and ligamento-genesis pathways [26,32,33]. Therefore, PDL stem cells *in vivo* are often stimulated to osteogenesis when influenced by physical and biochemical cues in certain physiological or pathological changes [34]. Damaged alveolar bone tissue proximity to PDL can thus be rapidly regenerated by the direct differentiation of PDL stem cells [35]. Of course the primary role of PDL cells is the maintenance of PDL structure and the regenerative actions of ligament structure when it was diseased and damaged [3]. Therefore, PDL tissue can be thought to be in the structural equilibrium with adjacent alveolar bone where the PDL stem cells are critically involved in the repair and regenerative processes of both tissues.

In this manner, we examined if the *in vitro* nanotopological and mechanical stimuli might have influenced the PDL cell differentiation preference, either to osteo- or ligamento-genesis. First, the ALP activity, a representative early osteogenic marker, has been used as the sign of PDL cellular osteogenesis. Our finding indicated the stress-free static cultures have significantly stimulated osteogenesis of PDL cells when cultured on both aligned and random nanofibers—in other words, dynamic mechanical stress down-regulated the osteogenic differentiation potential of the PDL cells. On the other hand, periostin and tenascin, the key PDL markers, were significantly up-regulated by the mechanical stimuli, and the alignment topological signal accelerated the stimulation, which was evidenced at the protein secretion level—in other words, the static culture on random nanofiber showed the least ligamentogenesis stimulation. Periostin, a key PDL protein in the fasciclin family, is known to form Sharpey’s fibers, the typical hierarchical PDL structure [36]. Periostin is preferentially expressed in the periosteum and PDL, which are continually subjected to mechanical stimuli, and mediate mesenchymal cell migration/invasion and matrix condensation [37]. It is also known to importantly regulate fibroblastic differentiation in regions that have the capacity to form bone [6]. Tenascin-C, a key marker for tendon/ligament fibroblasts, is an anti-adhesive protein and increases tissue elasticity in response to heavy loading [38]. Tenascin-C is expressed highly during development at the insertion sites of ligaments and tendons to bone and is localized immediately at the surrounding of cells [29]. When tenascin is strongly expressed in the PDL, it weakens the strong interaction of fibronectin with cells, up-regulating the expression of matrix metalloproteinases [28,39]. Moreover, tenascin allows better migration of PDL in response to mechanical stimuli by loosely packed cross-striated fibrils [40].Therefore, the significant stimulation of periostin and tenascin-C is indicative of the important role of the mechanical stimulus and nanotopological aligning in preserving the potential of PDL stem cells for ligamentogenesis and the possible remodeling of periodontal tissues under masticatory loading [28]. On the other hand, TGF-β1 was significantly stimulated only by the mechanical cues with being little affected by the nanofiber alignment. In fact, TGF-β1 is considered to be a potent osteogenic growth factor as well as a key inducer of proteins in the fasciclin family, thus is considered to regulate both osteo- and ligamento-genesis of multipotent cells [41,42]. TGF-β1 has been shown to be stimulated by mechanical forces to enhance matrix protein syntheses like osteopontin, which being also an essential marker of osteoblastic differentiation, provides anchoring sites for cells and mechanical elasticity and tension to bone matrix [42]. Furthermore, TGF-β1 increases the periostin gene expression in human PDL fibroblasts [43,44], and promotes the proliferation of human PDL cells while inhibiting osteoblast differentiation [44–46], suggesting it is a critical factor for the fiber formation in PDL tissue [43]. Taking all the above expression profiles of proteins (periostin, tenascin and TGF-β1) and ALP activity, the mechanical stress and nanotopological alignment have significant implications in improving PDL cell potential for ligamentogenesis as well as the possible engage in osteogenesis when needed. It should be born in mind that, in most cases, the *in vivo* regenerative conditions need ligamentogenesis, osteogenesis, and integration of ligament/bone interface at the same time, where the PDL stem cells should play decisive yet complicate roles [13]. Thus the current *in vitro* findings are considered to provide more relevant information on how PDL cells would behave under the dynamic mechanical-physical supported conditions that better mimic the *in vivo* environments [38].

We next designed *in vivo* models to test the feasibility of the tissue-engineered constructs in periodontal regeneration. After confirming the tissue compatibility in a rat subcutaneous model, premaxillary periodontal defect model was generated; first by the tooth extraction and replacement, disrupting the native interface of PDL/alveolar bone, and then either the critical-sized PDL/alveolar bone tissue being removed or remained intact. The removal model can demonstrate the ability to recover the severity and to regenerate neo-bone and -PDL tissues in defect, whereas the intact model informs how coherently the interfacial regeneration of PDL/alveolar bone will be.

Based on the micro-CT analyses and the histological findings, the mechanical stimulus and nanotopological aligning were demonstrated to play a synergistic role in improving the neo-bone formation. On the other hand, the mechanical loading had explicit additional effects on periodontal regeneration only in removal defect while the nanofiber alignment was effective for both intact and removal models. Conclusively, the effects of mechanical loading synergized with nanofiber alignment were revealed most significantly on the alveolar bone and PDL regeneration in removal model, whereas the nanofiber alignment effect was enough shown in the intact model. It is just thought that the clear evidence of the high potential of mechanically-cued PDL cells is revealed better in the regenerative conditions of tissues (either PDL or adjacent alveolar bone) that are severely damaged. However, the regenerated PDL structure in the removal defect even when tissue-engineered with mechanical and topological cues elicited non-oriented fibrous form, somewhat being deviated from the morphological feature of well-aligned compact native PDL tissue; therefore, future studies on the improvement of this issue will be needed.

## Conclusions

We applied cyclic mechanical loading on PDL cells upon topological cued nanofiber matrix using Flexcell system. The cellular protrusion and spreading shape were synergistically influenced by the mechanical and nanofiber alignment cues. The cues enabled PDL cells to undergo ligamentogenesis with simultaneous down-regulation of osteogenesis. *In vivo* periodontal defect study confirmed the mechanical- and topological-cued constructs had better regenerative capacity for hierarchical PDL structure as well as the adjacent alveolar bone tissue. This study provides informative idea for the regeneration of tissues including PDL, where topological and mechanical cues are both importantly considered.

## Supporting Information

S1 Table Tensile mechanical properties of nanofibers including elastic modulus, maximum stress and elongation. Data are presented as a mean (std. dev).
(DOCX)
