# Correction: Dynamic mechanical and nanofibrous topological combinatory cues designed for periodontal ligament engineering

**DOI:** 10.1371/journal.pone.0228475

**Published:** 2020-01-24

**Authors:** Joong-Hyun Kim, Min Sil Kang, Mohamed Eltohamy, Tae-Hyun Kim, Hae-Won Kim

An additional affiliation is missing for the third author. Mohamed Eltohamy is also affiliated with Department of Glass Research, National Research Centre, Dokki, Cairo, Egypt.
