# Supplementary material for: Dynamic Mechanical and Nanofibrous Topological Combinatory Cues Designed for Periodontal Ligament Engineering
Source: PLoS One. 2016 Mar 18;11(3):e0149967. doi: 10.1371/journal.pone.0149967 (PMC4798756; doi:10.1371/journal.pone.0149967)
Supplement: S1 Table — Data are presented as a mean (std. dev). (DOCX) [file pone.0149967.s001.docx]

Supporting Table 1. Tensile mechanical properties of nanofibers including elastic modulus, maximum stress and elongation. Data are presented as a mean (std. dev).

|  | aligned | random |
| --- | --- | --- |
| Elastic modulus (kPa) | 3330.1 (691.5) | 1357.9 (250.9) |
| Maximum stress (kPa) | 586.9 (66.4) | 1557.3 (369.0) |
| Elongation % | 68.4 (23.6) | 292.5 (31.7) |
